# A Case in Proof of Non-Identity of Variola and Varicella

**Published:** 1878-01

**Authors:** Seymour J. Sharkey

**Affiliations:** Resident Assistant Physician. St. Thomas’ Hospital


### A Case in Proof of Non-Identity of Variola and Varicella.

By Seymour J. Sharkey, Resident Assistant Physician. St. Thomas' Hospital.

Thomas B——, aged five years, left the scarlet fever ward of St. Thomas's Hospital on Nov. 22d, 1876, where he had been under the care of Dr. Bristowe. In the same block, and on the floor above, there were during the child's stay in the hospital, several cases of small-pox. Since his discharge from the hospital he had never felt quite well, but had suffered from headache, slight pain in the back, and anorexia. His friends brought him back on Nov. 28th, with a vesicular eruption upon him, which was said to have appeared first on the legs, though it was then most profuse on the abdomen and back. The child was stated never to have had chicken-pox, and never to have been vaccinated, and there were no marks of vaccination upon him.

On admission, the eruption was not well enough developed to to make a certain diagnosis between varicella and variola, and he was therefore isolated in a small ward on the same floor as the small-pox wards. Soon, however, the disease showed all the characters of varicella, and was pronounced to be so by Dr. Risdon Bennett and Dr. Bristowe. The patient was then removed to the floor below, and during the next few days fresh crops of vesicles appeared, which were vesicular from the first, had no induration around them, and dried up rapidly.

As small-pox was rife in London, and there were cases of the disease in the block, it was thought advisable to vaccinate the child at once. This was done in four places on Nov. 30th. Four very fine vesicles resulted, which ran their normal course at first, but the areola was never properly developed round them. On December 7th—that is, on the eighth day from vaccination—the child became very restless, his sleep was much disturbed by dreams and apparitions; he had pain in the back, vomited several times, and was feverish. The vomiting was frequent and violent on the following day, and on the 9th of December a few papules appeared on the face, then on the arms; and on the 11th the face, arms, legs, back, and abdomen showed a profuse crop of papules which were clearly the early eruption of small-pox, the scabs of varicella being still present. The primary fever was very considerable, the temperature reaching 105° F., but as soon as the eruption appeared it dropped to 99°. The eruption developed in the ordinary way, and secondary fever of moderate intensity set in early, as shown in the temperature chart. The eruption was profuse, but not confluent. Convalescence was very tedious, and was interrupted by an enormous, hard swelling on the left side of the neck beneath the lower jaw, which appeared to commence in the lymphatic glands, and subsequently suppurated. The child recovered however, and was discharged from the hospital on February 10th, 1877.

This is a case of considerable importance, and one that should be put on record, and the paper recently read by Dr. Farquharson before the Clinical Society has led Dr. Bristowe to give me permission to publish it at once. It places beyond doubt the non-identity of varicella and variola, and shows that vaccination does not prevent the incubating poison of small-pox from producing a well-developed attack of the disease, although eight days have elapsed from the time of the operation. It also shows that an individual may harbor at the same time the poisons of two infectious diseases, or at at any rate the poisons of varicella and variola, each of which shall in due time produce a well-marked attack of the disease in which it originated. For, if we take twelve days as the usual period of the incubation of small-pox, and ten and sixteen days (Bristowe) as the extremes, the patient in question must have been infected by the poison of variola either when the eruption of varicella was out or during the time of incubation of that disease. —*London Lancet.*

